# Supplementary material for: HIV-1 vif mediates ubiquitination of the proximal protomer in the APOBEC3H dimer to induce degradation
Source: Nat Commun. 2025 Jul 1;16:5879. doi: 10.1038/s41467-025-60984-y (PMC12217271; doi:10.1038/s41467-025-60984-y)
Supplement: Supplementary file 1 — Supplementary Information [file 41467_2025_60984_MOESM1_ESM.pdf]

Title: **HIV-1 Vif Mediates Ubiquitination of the Proximal Protomer in the APOBEC3H Dimer to Induce Degradation**

Supplementary Table 1: Oligonucleotides used in this study

Oligonucleotide Name	Oligonucleotide DNA sequence (5' ->3')	
Myc-A3H ATG(+)	GTCTTTTCTGCAGTCACCGTCCAAGCTTCGATGGAGCAGAAACTCATCTCTGAAGAGGATCTGGGGATGGCTCTGTTAACAGCCGAAA	pTR600 myc-cpzA3H WT
APO3H Stop AvrII (-)	ACAGCCTAGGTCAGGACTGCTTTATCCT	
Fwd_PstI&HindIII_cpzA3H	GTCTTTTCTGCAGTCACCGTCCAAGCTTCGATGGCTCTGTTAACAGCCGAAA	pTR600 cpzA3H WT (no tag)
APO3H Stop AvrII (-)	ACAGCCTAGGTCAGGACTGCTTTATCCT	
HVif Y30A (+)	CCACATGGCCATTAGCCGCAAAGCTAAG	HVif Y30A
HVif Y30A (-)	GCTAATGGCCATGTGGTGCTTCACCAG	
HVif K36A (+)	CCACATGTACATTAGCCGCAAAGCTGCGGACTGGTTCTACCGC	HVif K36A
BGH Reverse Primer	TAGAAGGCACAGTCGAGGCTG	
HVif F39A (+)	CCACATGTACATTAGCCGCAAAGCTAAGGACTGGGCCTACCGCCACCACTAC	HVif F39A
BGH Reverse Primer	TAGAAGGCACAGTCGAGGCTG	
HVif R41A (+)	CCACATGTACATTAGCCGCAAAGCTAAGGACTGGTTCTACGCCCACCACTACGAGAGC	HVif R41A
BGH Reverse Primer	TAGAAGGCACAGTCGAGGCTG	
HVif E45A (+)	CCACATGTACATTAGCCGCAAAGCTAAGGACTGGTTCTACCGCCACCACTACGCGAGCACCAACCCCAAGA	HVif E45A
BGH Reverse Primer	TAGAAGGCACAGTCGAGGCTG	
HVif H56A (+)	CCACATGTACATTAGCCGCAAAGCTAAGGACTGGTTCTACCGCCACCACTACGAGAGCACCAACCCCAAGATTAGCAGCGAGGTAGCCATTCCCCTGGGCGACGCCA	HVif H56A
BGH Reverse Primer	TAGAAGGCACAGTCGAGGCTG	
T7 Promoter	TAATACGACTCACTATAGGG	HVif K63A
HVif K63A (-)	CCCTGGCCAAGGTGCCAGTCGCGCTCGCCGGTGTGCAGGCCCCAGTAGGTCGTAATCACCAGCGCGGCGTCGCCCAGGGGAAT	
HVif_K92A(+)	CAGGGCGTCTCCATAGAATGGAGGAAAGCCAGATATAGCACACAAGTA	HVif K92A
HVif_E88(-)	TTCTATGGAGACGCCCTGGCCCAG	
Hvif_R93A(+)	CAGGGCGTCTCCATAGAATGGAGGAAAAAGGCCTATAGCACACAAGTAGAC	HVif R93A
HVif_E88(-)	TTCTATGGAGACGCCCTGGCCCAG	
HVif H42A (+)	CCACATGTACATTAGCCGCAAAGCTAAGGACTGGTTCTACCGCGCCCACTACGAGAGCACC	HVif H42A
BGH Reverse Primer	TAGAAGGCACAGTCGAGGCTG	
HVif H43A (+)	CCACATGTACATTAGCCGCAAAGCTAAGGACTGGTTCTACCGCCACGCCTACGAGAGCACCAAC	HVif H43A
BGH Reverse Primer	TAGAAGGCACAGTCGAGGCTG	
HVif Y44A (+)	CCACATGTACATTAGCCGCAAAGCTAAGGACTGGTTCTACCGCCACCACGCCGAGAGCACCAACCCC	HVif Y44A
BGH Reverse Primer	TAGAAGGCACAGTCGAGGCTG	
HVif P49A (+)	CACATGTACATTAGCCGCAAAGCTAAGGACTGGTTCTACCGCCACCACTACGAGAGCACCAACGCCAAGATTAGCAGCGA	HVif P49A
BGH Reverse Primer	TAGAAGGCACAGTCGAGGCTG	
T7 Promoter	TAATACGACTCACTATAGGG	HVif W70A
HVif W70A (-)	CCCTGGCCAAGGTGCCAGTCGCGCTCGCCGGTGTGCAGGCCTGCGTAGGTCGTAATCACCAG	
Hvif_H80A(+)	CACACCGGCGAGCGCGACTGGGCCCTGGGCCAGGGCGTCTCC	HVif H80A
Hvif_W79(-)	CCAGTCGCGCTCGCCGGTGTGCAG	
T7 Promoter	TAATACGACTCACTATAGGG	HVif R23A
HVif R23A (-)	GCTAATGTACATGTGGTGCTTCACCAGAGCCTTCCAGGTGTTAATGCG	
T7 Promoter	TAATACGACTCACTATAGGG	HVif H27A
HVif H27A (-)	GCTAATGTACATGTGGGCCTTCACCAGGCGCTTCCAGGT	
HVif_K160(+)	AAGCCACCTTTGCCTAGTGTTAGG	HVif K157A
HVif_K157A(-)	ACTAGGCAAAGGTGGCTTTATCTGGGCTGGTTTTATTAATGCTGC	
T7 Promoter	TAATACGACTCACTATAGGG	HVif K160A
HVif K160A (-)	CCTAACCCTAGGCAAAGGTGGCGCTATCTGTTTTGGTTTTAT	
T7 Promoter	TAATACGACTCACTATAGGG	HVif P161A
HVif P161A (-)	CCTAACCCTAGGCAAAGGTGCCTTTATCTGTTTTGGTTTTAT	
HVif R15A (+)	TGCAGAATTCATGGAGAACCGGTGGCAGGTGATGATTGTGTGGCAGGTGGACGCCATGCGCATTAACACCTGGAAGCGC	HVif R15A
BGH Reverse Primer	TAGAAGGCACAGTCGAGGCTG	
Hvif_L81A(+)	CACACCGGCGAGCGCGACTGGCACGCTGGCCAGGGCGTCTCCATA	HVif L81A
Hvif_W79(-)	CCAGTCGCGCTCGCCGGTGTGCAG	
Fwd_cpzA3H_R27K	CCGAGGAAGGCCCTCTTGTGTTACCAGCTGACGCC	7KR+R27K
Rev_cpzA3H_R27K	GAGGGCCTTCCTCGGGTAGTAAGGCCTTCTGAGGC	
Fwd_cpzA3H_R50K	GAAAACAAGAGAAGGTGCCATGCAGAAATTTGCTT	7KR+R50K
Rev_cpzA3H_R50K	CCTTCTCTTGTTTTCAAAGTAGCCTCTGGTGGGCG	
Fwd_cpzA3H_R51K	AACAGGAAAAGGTGCCATGCAGAAATTTGCTTTAT	7KR+R51K
Rev_cpzA3H_R51K	GCACCTTTTCCTGTTTTCAAAGTAGCCTCTGGTGG	
Fwd_cpzA3H_R52K	AGGAGAAAGTGCCATGCAGAAATTTGCTTTATTAA	7KR+R52K
Rev_cpzA3H_R52K	ATGGCACTTTCTCCTGTTTTCAAAGTAGCCTCTGG	
Fwd_cpzA3H_R153K	CACGAGAAACCGCTTTCCTTCGACCCCTGTCGCAT	7KR+R153K
Rev_cpzA3H_R153K	AAGCGGTTTCTCGTGGTCCACAAAGTTTTCCCAGC	
Fwd_cpzA3H_R161K	CCCTGTAAGATGTTAGAGGAGCTAGATCGCAACAG	7KR+R161K
Rev_cpzA3H_R161K	TAACATCTTACAGGGGTCGAAGGAAAGCGGGCGCT	
Fwd_cpzA3H_R168K	CTAGATAAAAACAGTCGAGCCATAAAGCGACGGCT	7KR+R168K
Rev_cpzA3H_R168K	ACTGTTTTTATCTAGCTCCTCTAACATGCGACAGG	

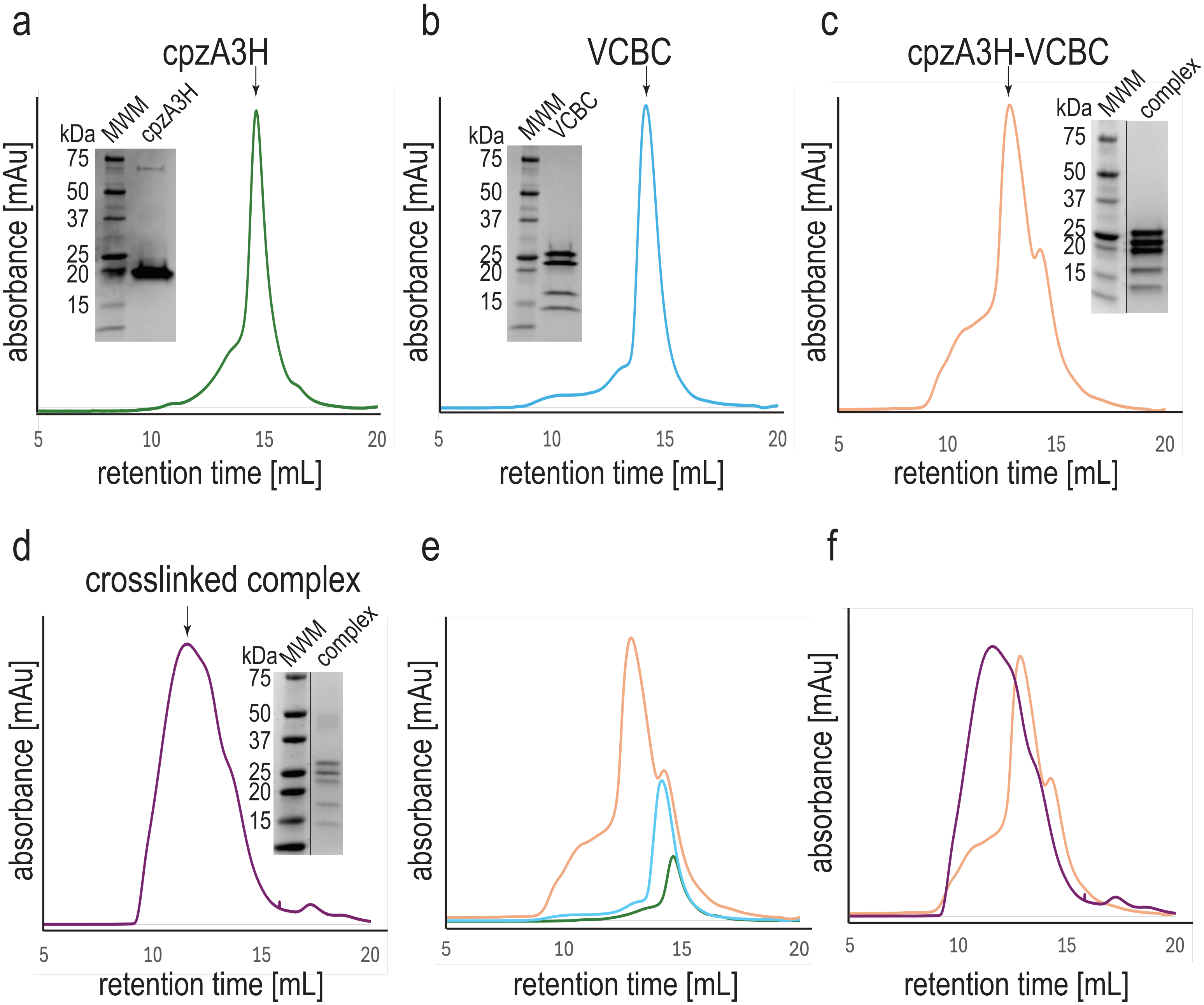

a
cpzA3H
b
VCBC
c
cpzA3H-VCBC
d
crosslinked complex
e
f
kDa
MWM
cpzA3H
VCBC
complex
75
50
37
25
20
15
absorbance [mAu]
retention time [mL]
5
10
15
20

Supplementary Figure 1– ***Purification of the cpzA3H-VCBC complex.***

Size-exclusion chromatograms and SDS-PAGE analyses of the cpzA3H-VCBC complex formation. (**a**) Size-exclusion chromatogram of purified cpzA3H, with an inset showing Coomassie-stained SDS-PAGE of the peak fraction (indicated by arrow). (**b**) Chromatogram of the purified VCBC complex, with peak fraction (arrow) shown in the SDS-PAGE inset. (**c**) Chromatogram of a 1:1 molar mixture of cpzA3H and VCBC, with an inset showing SDS-PAGE of the peak fraction (arrow) indicating all five proteins corresponding to the cpzA3H-VCBC complex. Peak fractions were pooled, concentrated, and subjected to crosslinking. (**d**) Chromatogram of the crosslinked cpzA3H-VCBC complex, with the peak fraction (arrow) used for cryo-EM, and corresponding SDS-PAGE showing all five proteins. (**e**) Superimposed chromatograms from (**a**), (**b**), and (**c**), indicating cpzA3H alone (green), VCBC alone (blue), and the cpzA3H-VCBC complex (orange). (**f**) Superimposed chromatograms from (**c**) and (**d**), showing the cpzA3H-VCBC complex before and after BS3 crosslinking, in orange and purple, respectively.

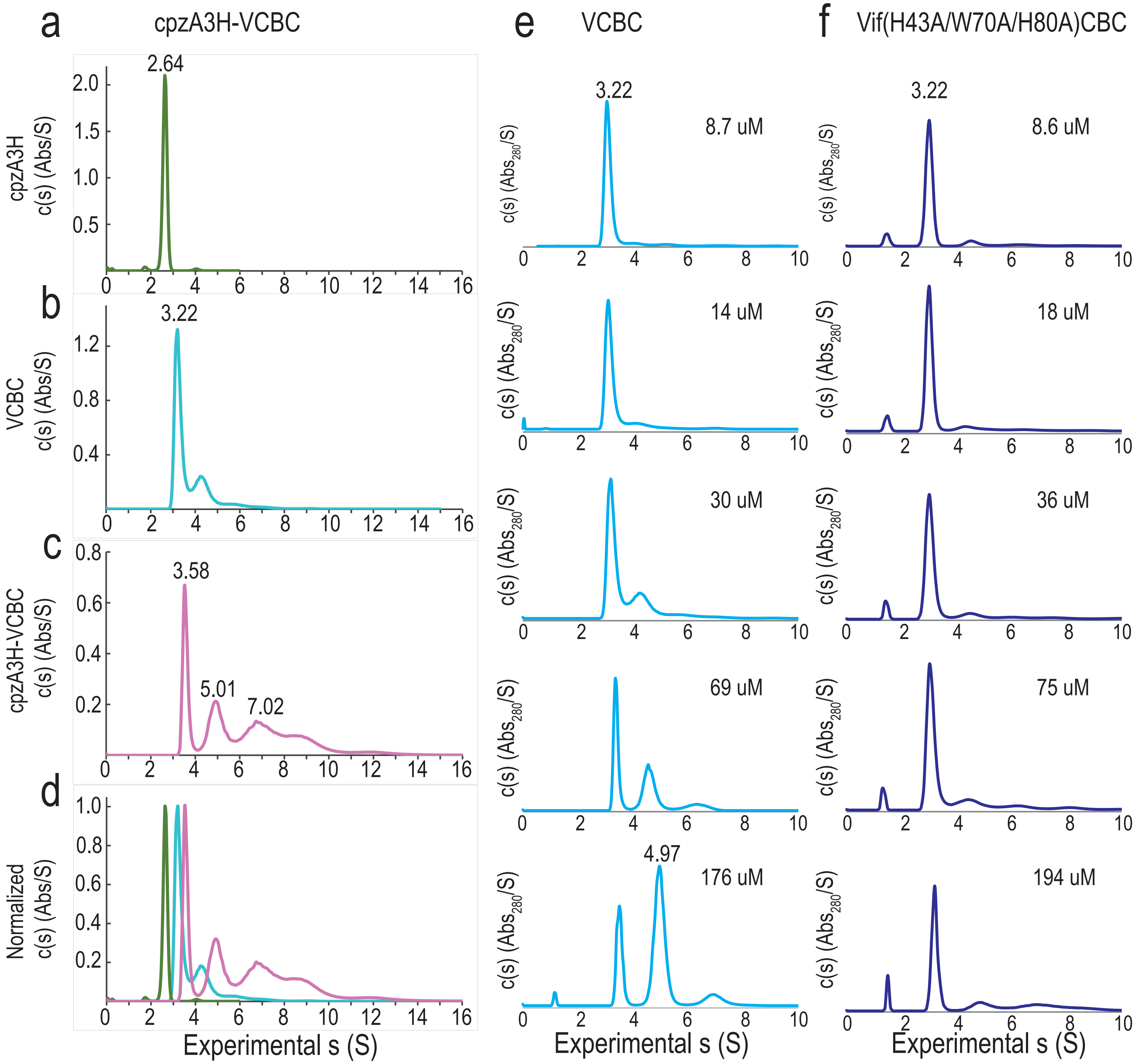

a
cpzA3H-VCBC
cpzA3H
c(s) (Abs/S)
2.64
b
VCBC
c(s) (Abs/S)
3.22
c
cpzA3H-VCBC
c(s) (Abs/S)
3.58
5.01
7.02
d
Normalized
c(s) (Abs/S)
Experimental s (S)
e
VCBC
3.22
8.7 uM
14 uM
30 uM
69 uM
4.97
176 uM
c(s) (Abs280/S)
Experimental s (S)
f
Vif(H43A/W70A/H80A)CBC
3.22
8.6 uM
18 uM
36 uM
75 uM
194 uM
c(s) (Abs280/S)
Experimental s (S)

Supplementary Figure 2 - ***Sedimentation velocity analytical ultracentrifugation of the cpzA3H-VCBC complex***

Panel **a:** Sedimentation velocity c(s) profile for cpzA3H shows a species at 2.64 S with an estimated molar mass of 52 kDa, corresponding to the cpzA3H dimer, composed of two cpzA3H molecules bound to a dsRNA. Panel **b:** Sedimentation velocity c(s) profile for VCBC at 30 μM shows a species at 3.22 S, corresponding to the monomeric VCBC complex, along with species at higher S values, indicating the presence of fast exchanging VCBC dimers and higher states. At 4 μM, the VCBC complex shows a single species at 3.03 S and 64 kDa, corresponding to the monomeric complex (data not shown). Panel **c:** Sedimentation velocity c(s) profile for an equimolar mixture of cpzA3H and VCBC, indicating a dynamic exchange between free cpzA3H and free VCBC, the monomeric cpzA3H-VCBC complex, the dimeric cpzA3H-VCBC complex, and higher-order multimers. The slow sedimenting species at 3.58 S represents free VCBC, whereas the fast species at 5.01 S and 7.02 S represent the reaction boundaries for the monomeric and dimeric cpzA3H-VCBC complexes, respectively. Panel **d** overlays the normalized data from panels **a**, **b**, and **c** for direct comparison. Panel **e:** Sedimentation velocity c(s) profiles of VCBC across increasing protein concentrations (8.7 μM to 176 μM) show a shift from a species at 3.22 S, corresponding to the monomeric VCBC complex, to species at 4.97 S and higher S values, indicating the presence of rapidly exchanging VCBC dimers and higher-order oligomers. Panel **f:** Sedimentation velocity c(s) profiles of VCBC containing the Vif(H43A/W70A/H80A) variant at increasing concentrations (8.6 μM to 194 μM) reveal a single species at 3.22 S, consistent with the monomeric VCBC complex. The absence of higher S-value species across all concentrations indicates that dimerization or higher-order oligomerization is not significant under these conditions.

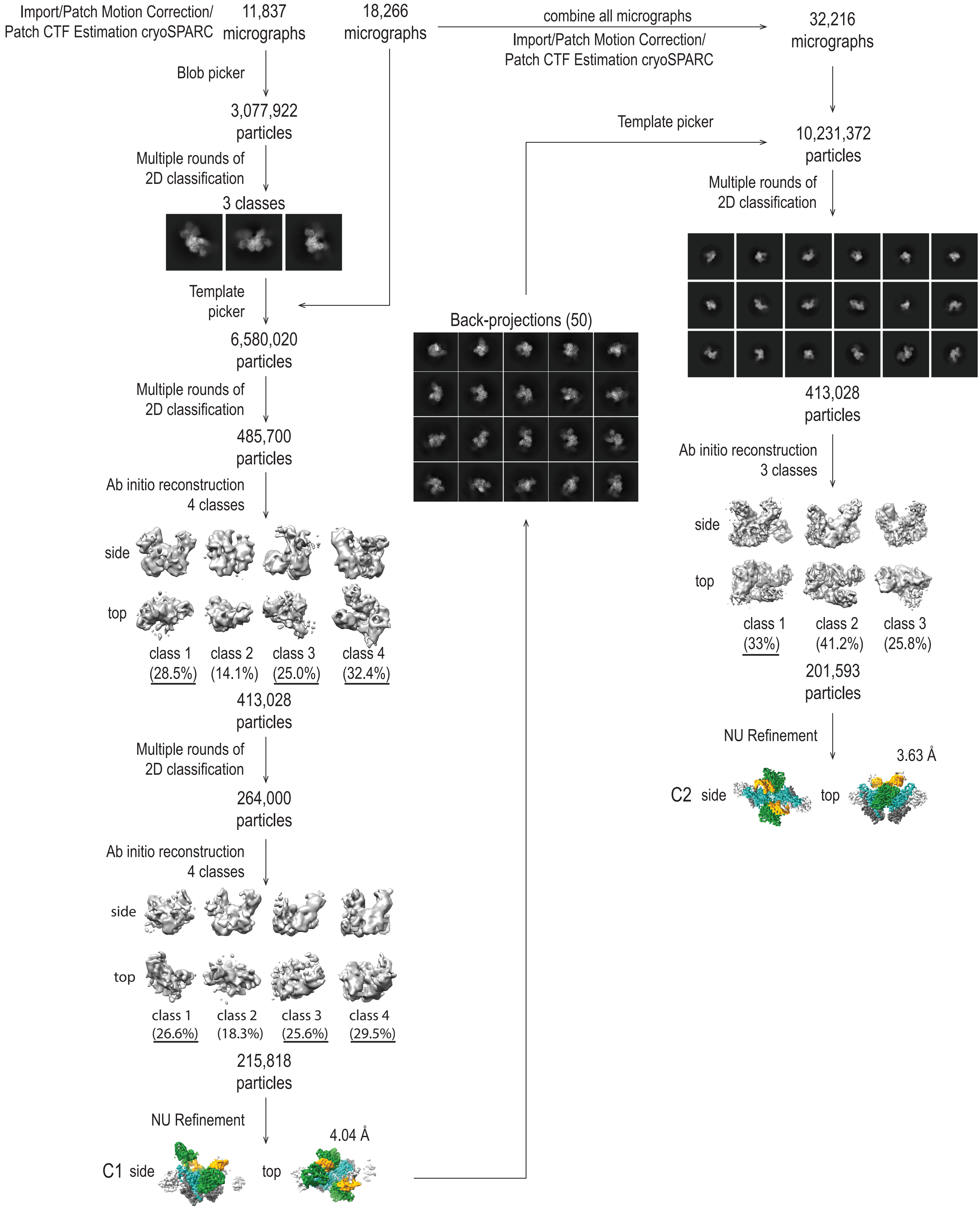

Import/Patch Motion Correction/
Patch CTF Estimation cryoSPARC
11,837
micrographs
18,266
micrographs
combine all micrographs
Import/Patch Motion Correction/
Patch CTF Estimation cryoSPARC
32,216
micrographs
Blob picker
3,077,922
particles
Multiple rounds of
2D classification
3 classes
Template
picker
6,580,020
particles
Multiple rounds of
2D classification
485,700
particles
Ab initio reconstruction
4 classes
side
top
class 1
(28.5%)
class 2
(14.1%)
class 3
(25.0%)
class 4
(32.4%)
413,028
particles
Multiple rounds of
2D classification
264,000
particles
Ab initio reconstruction
4 classes
side
top
class 1
(26.6%)
class 2
(18.3%)
class 3
(25.6%)
class 4
(29.5%)
215,818
particles
NU Refinement
4.04 Å
C1 side
top
Back-projections (50)
Template picker
10,231,372
particles
Multiple rounds of
2D classification
413,028
particles
Ab initio reconstruction
3 classes
side
top
class 1
(33%)
class 2
(41.2%)
class 3
(25.8%)
201,593
particles
NU Refinement
3.63 Å
C2 side
top

Supplementary 3– ***Cryo-EM image processing workflow*****.**
Movie data were collected using K3 direct detector in counting mode, on Talos Arctica operating at the acceleration voltage of 200 kV. Initially, 3,077,922 particles were collected from 11,834 motion-corrected, dose-weighted micrographs. 3 top 2D classes were used to obtain 6,580,020 particles from 30,103 motion-corrected, dose-weighted micrographs. 485,700 particles were used for ab-initio 3D reconstruction. After 2D/3D classification, 215,818 particles were selected for the 3D refinement, without symmetry applied. The resolution of obtained map was estimated at 4.0 Å, based on Fourier Shell Correlation (FSC) = 0.143. The final C1 map was used to generate 50 2D projections that were than used as initial reference to re-process all data. After 2D/3D classification, 201,593 particles were selected for the 3D refinement, with C2 symmetry applied. The resolution of obtained map was estimated at 3.6 Å, based on FSC = 0.143. Both final maps were subjected to post-processing with EMready[8] to improve their interpretability.

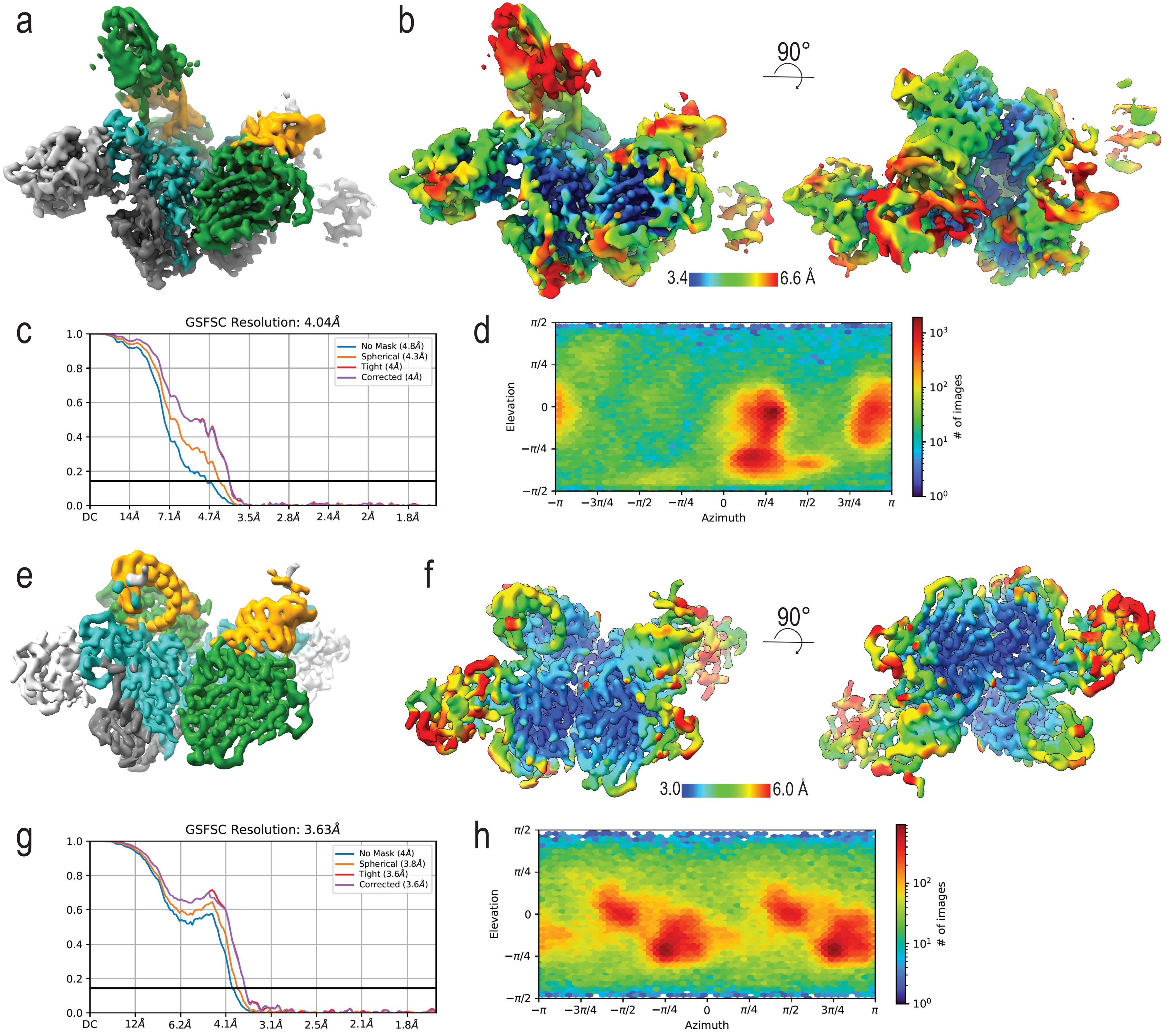

a
b
90°
3.4
6.6 Å
c
GSFSC Resolution: 4.04Å
No Mask (4.8Å)
Spherical (4.3Å)
Tight (4Å)
Corrected (4Å)
DC
14Å
7.1Å
4.7Å
3.5Å
2.8Å
2.4Å
2Å
1.8Å
d
Elevation
Azimuth
# of images
e
f
90°
3.0
6.0 Å
g
GSFSC Resolution: 3.63Å
No Mask (4Å)
Spherical (3.8Å)
Tight (3.6Å)
Corrected (3.6Å)
DC
12Å
6.2Å
4.1Å
3.1Å
2.5Å
2.1Å
1.8Å
h
Elevation
Azimuth
# of images

Supplementary Figure 4- ***Resolution evaluation of the cpzA3H-VCBC complex*****.**
Evaluation of the C1 (**a-d**) and C2 (**e-h**) models of the cpzA3H-VCBC complex including Cryo-EM density map (**a, e**), local resolution shown on the density map (**b, f**), global resolution estimation based on the gold standard Fourier shell correlation (FSC) coefficient of 0.143 criterion (**c, g**), and angular distribution plot of the particles (**d**, **h**). In panels (a) and (e), cpzA3H, CBFβ, Vif, and Elongin B & C are colored green, dark gray, cyan, and light gray, respectively.

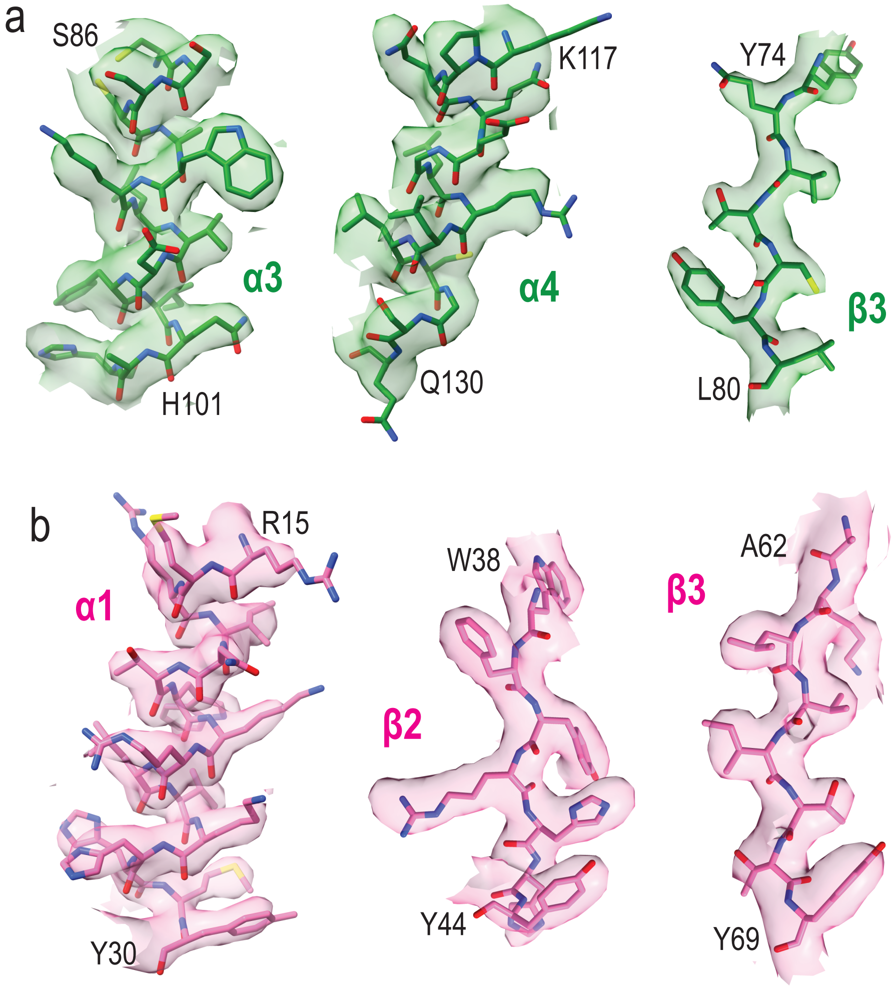

a
S86
K117
Y74
α3
α4
β3
H101
Q130
L80
b
R15
α1
W38
A62
β3
β2
Y30
Y44
Y69

Supplementary Figure 5 – *Model to map fit.*
The representative secondary structures of cpzA3H (**a**) and Vif (**b**) molecules are shown with the corresponding cryo-EM densities.

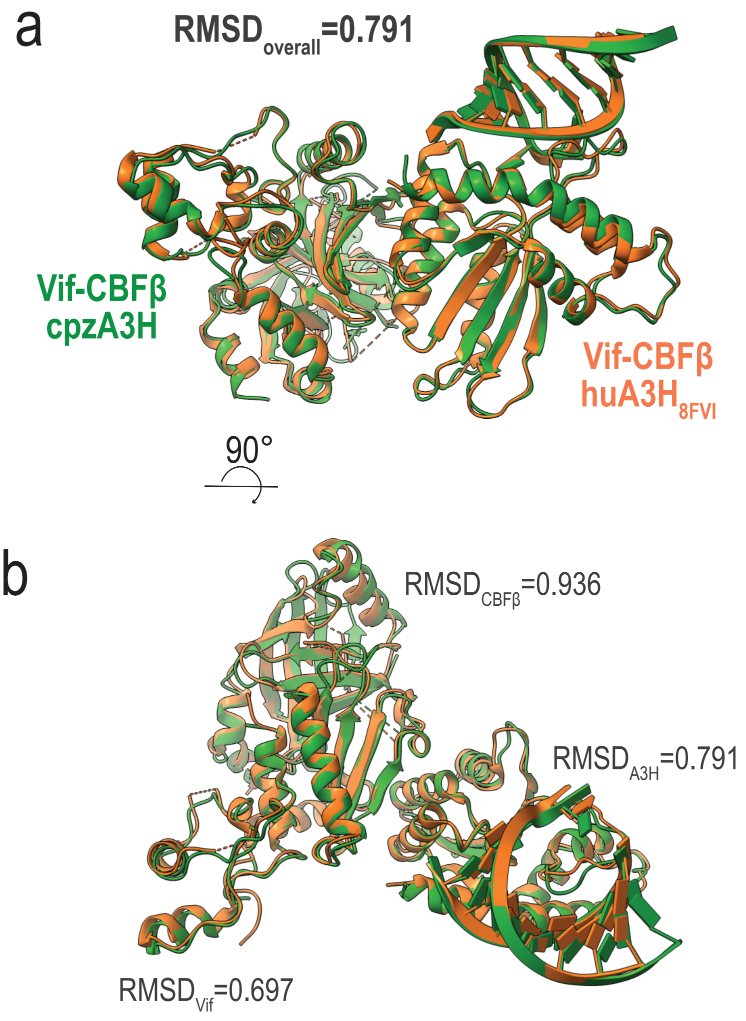

c Comparison of hA3H haplotype II and cpzA3H amino acids at the interface with Vif

```
cpzA3H  1    MALLTAETFRLQFNNRRRLRRPYYPRKALLCYQLTPQNGSTPTRGYFENKKKCHAEICFI  60
hA3H    1    MALLTAETFRLQFNNKRRLRRPYYPRKALLCYQLTPQNGSTPTRGYFENKKKCHAEICFI  60

cpzA3H  61   NEIKSMGLDETQCYQVTCYLTWSPCSSCAWKLVDFIQAHDHLNLRIFASRLYYHWCKPQQ 120
hA3H    61   NEIKSMGLDETQCYQVTCYLTWSPCSSCAWELVDFIKAHDHLNLRIFASRLYYHWCKPQQ 120

cpzA3H  121  EGLRLLCGSQVPVEVMGLPEFNDCWENFVDHEKPLSFDPCKMLEELDKNSRAIKRRLERI 180
hA3H    121  DGLRLLCGSQVPVEVMGFPEFADCWENFVDHEKPLSFNPYKMLEELDKNSRAIKRRLDRI 180

cpzA3H  181  KQS 183
hA3H    181  K-S 182
```

Supplementary Figure 6: *Structural comparison with the hA3H-VCBC structure*
(a, b) Structural comparison of cpzA3H–Vif–CBFβ (this study, dark green) and humanA3H–Vif–CBFβ (PDB: 8FVI[9], orange) complexes. RMSD values between the two cryo-EM structures, as well as between individual component proteins, are indicated.
(c) Sequence alignment of cpzA3H and hA3H. In cpzA3H, residues that interact with Vif from the same protomer are colored cyan, while those interacting with Vif from the second protomer are colored pink. In hA3H, Vif-interacting residues are colored brown. Residues involved in Vif binding were identified in cpzA3H–Vif–CBFβ and hA3H–Vif–CBFβ structures using the *InterfaceResidues* script from the PyMOLWiki (https://www.pymolwiki.org/index.php/InterfaceResidues).

a

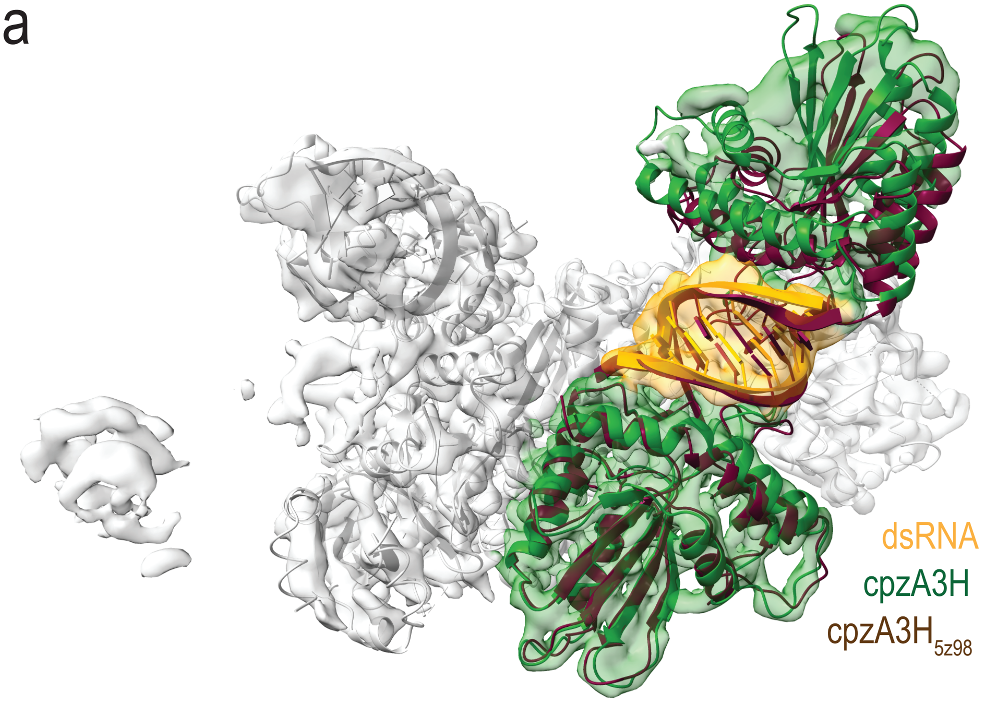

b

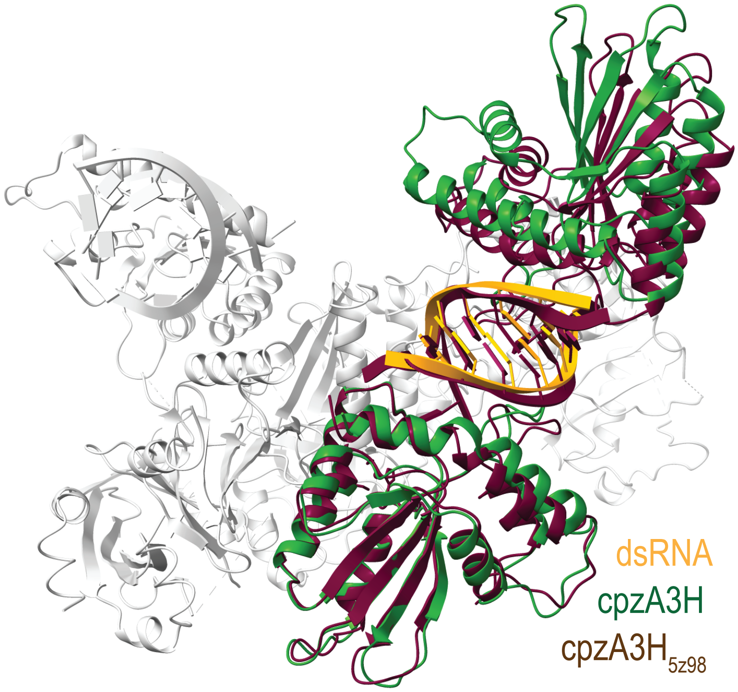

Supplementary Figure 7. - ***Structural Comparison of the Cryo-EM Model to the Crystal Structure of A3H.***

Superimposition of the cpzA3H crystal structure (brown, PDB ID#5Z98)[10] and cryo-EM cpzA3H structure from the C1 map of cpzA3H-VCBC complex (this study, cpzA3H:green, dsRNA: yellow), with (**a**) and without (**b**) the EM map.

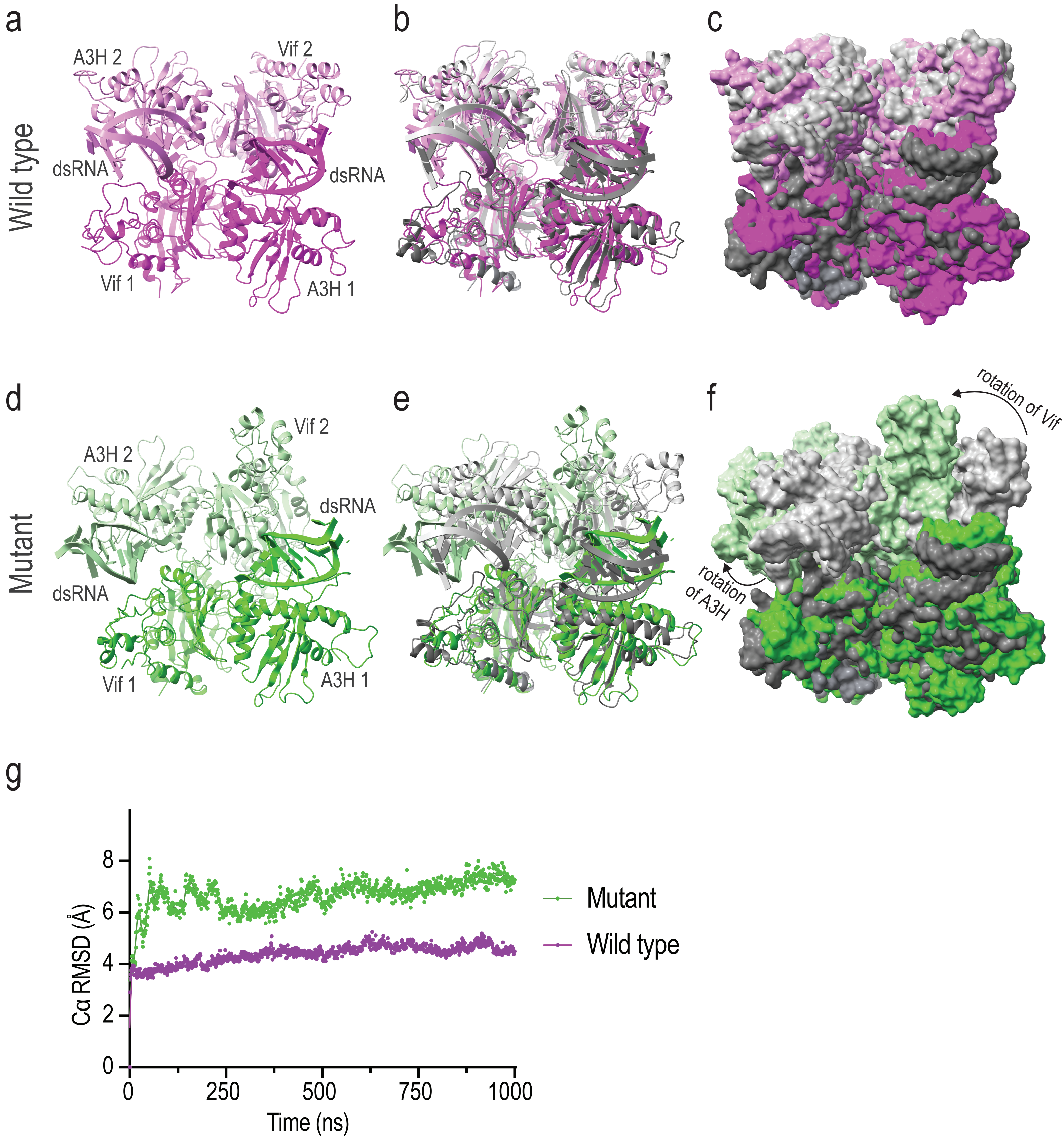

a
b
c
Wild type
A3H 2
Vif 2
dsRNA
dsRNA
Vif 1
A3H 1
d
e
f
Mutant
Vif 2
A3H 2
dsRNA
dsRNA
Vif 1
A3H 1
rotation of Vif
rotation of A3H
g
Cα RMSD (Å)
Time (ns)
0
2
4
6
8
0
250
500
750
1000
Mutant
Wild type

Supplementary Figure 8 – *MD simulation*.

Protomer dimerization demonstrates stability in molecular simulations. Shown is the ribbon diagram of the WT (**a**) and the H43A/W70A/H80A mutant (**d**) dimer structures after 1000 ns of molecular dynamics. Superimpositions of ribbon diagrams of the initial model and the structure following 1000 ns molecular dynamics for the WT dimer are illustrated in (**b**), along with their surface representations in (**c**). To highlight the relative movement, the cpzA3H-Vif from individual protomers are superimposed, showing the positional shift of the other protomers. In (**e**) and (**f**), the ribbon diagram and surface representations, respectively, of the H43A/W70A/H80A mutant dimer are displayed, with cpzA3H-Vif of single protomer superimposed to emphasize the movement of the other protomer. The rotation of cpzA3H and Vif is indicated by arrows in panel (f). Darker shades in panels (**b**), (**c**), (**e**), and (**f**) denote the superimposed protomers. (**g**) illustrates the Cα RMSD variations for the WT and H43A/W70A/H80A mutant over time, with WT and mutant results shown in purple and green, respectively.

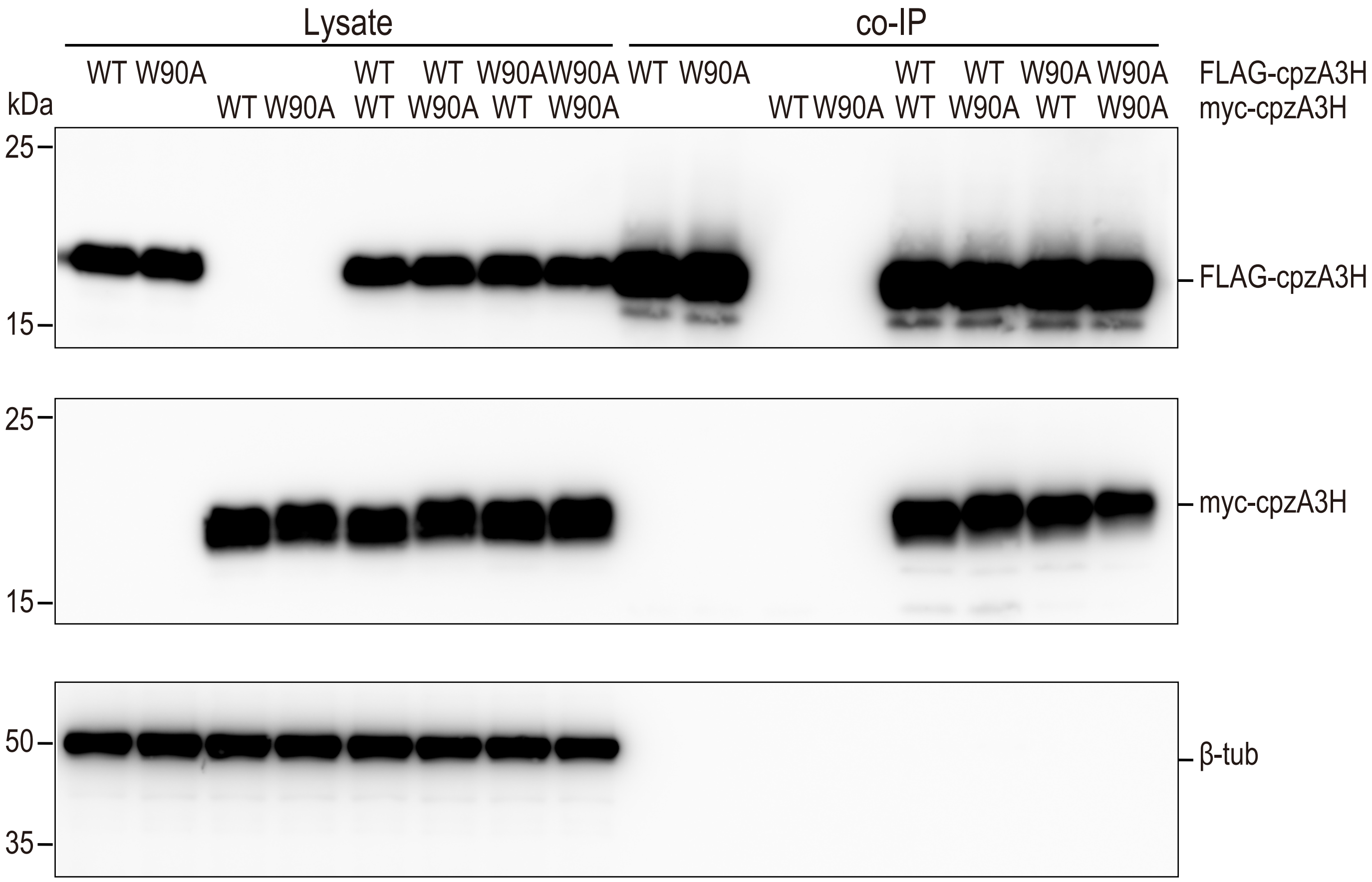
Lysate
co-IP
WT W90A WT W90A WT W90A WT W90A W90A W90A WT W90A WT W90A WT W90A WT W90A W90A W90A
FLAG-cpzA3H
myc-cpzA3H
kDa
25
15
25
15
50
35
FLAG-cpzA3H
myc-cpzA3H
β-tub

Supplementary Figure 9: ***Confirmation of heterodimer formation between WT and W90A variant cpzA3H proteins.***

FLAG-tagged and myc-tagged cpzA3H were co-expressed in HEK293T cells (lanes 5–8, 13–16). As controls, either FLAG-cpzA3H (lanes 1–2, 9–10) or myc-cpzA3H (lanes 3–4, 11–12) was expressed individually. FLAG-cpzA3H/myc-cpzA3H heterodimers were co-immunoprecipitated using anti-FLAG M2 beads and eluted with 3× FLAG peptide. Total cell lysates (Lysate) and immunoprecipitates (co-IP) were analyzed by immunoblotting with anti-FLAG and anti-myc monoclonal antibodies. Anti-β-tubulin polyclonal antibody (β-tub) was used as a loading control.

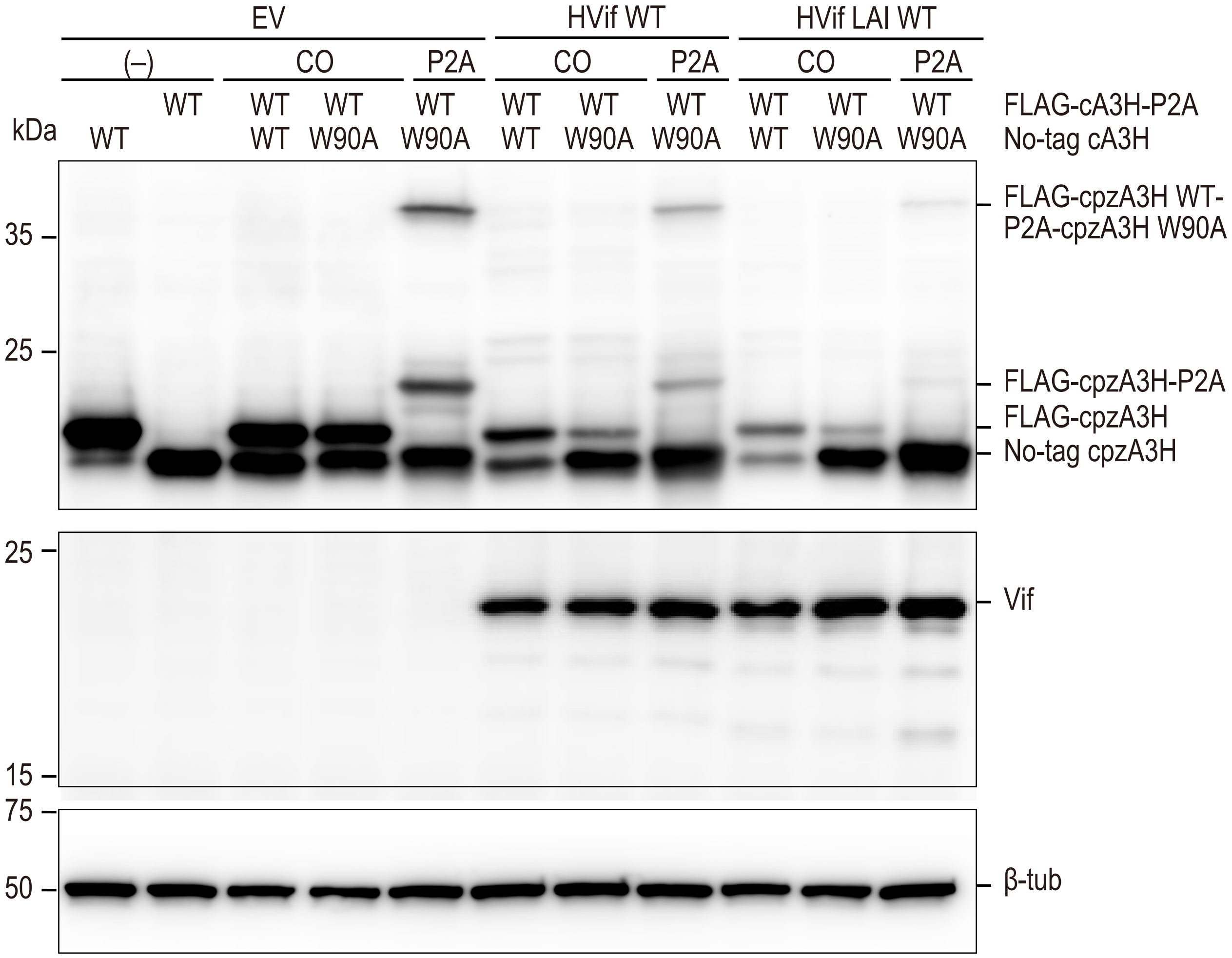
EV
HVif WT
HVif LAI WT
(–)
CO
P2A
CO
P2A
CO
P2A
WT
WT
WT
WT
WT
WT
WT
WT
WT
WT
WT
WT
WT
WT
W90A
W90A
WT
W90A
W90A
WT
W90A
W90A
FLAG-cA3H-P2A
No-tag cA3H
kDa
35
25
FLAG-cpzA3H WT-P2A-cpzA3H W90A
FLAG-cpzA3H-P2A
FLAG-cpzA3H
No-tag cpzA3H
25
15
Vif
75
50
β-tub

Supplementary Figure 10 - ***Analysis of the Vif-dependent degradation of the cpzA3H heterodimer proteins by using P2A polycistronic expression system.***

The degradation experiment used a single vector containing Flag-tagged wild-type cpzA3H, a P2A self-cleavage site, and W90A cpzA3H, generating Flag-tagged wild-type cpzA3H and untagged W90A cpzA3H from a single transcript. We co-transfected HEK293T cells with empty vector (no Vif) or the NL4-3 Vif WT (HVif WT) or LAI WT Vif expression plasmids, with the cpzA3H (WT or W90A) (no tag) and FLAG-cpzA3H WT expression plasmids, or FLAG-cpzA3H WT-P2A-cpzA3H W90A expression plasmid. At 40 h post-transfection, the intracellular A3H levels were compared by western blot analysis using an anti-A3H rabbit polyclonal antibody. The Vif expression levels were detected with an anti-Vif monoclonal antibody. The anti-ß tubulin polyclonal antibody (ß-Tub) was used as the loading control. CO: co-transfection, P2A: P2A polycistronic expression. Lanes 8 and 11 show that only wild-type cpzA3H is degraded, consistent with the results from the co-transfection experiments (lanes 7 and 10).

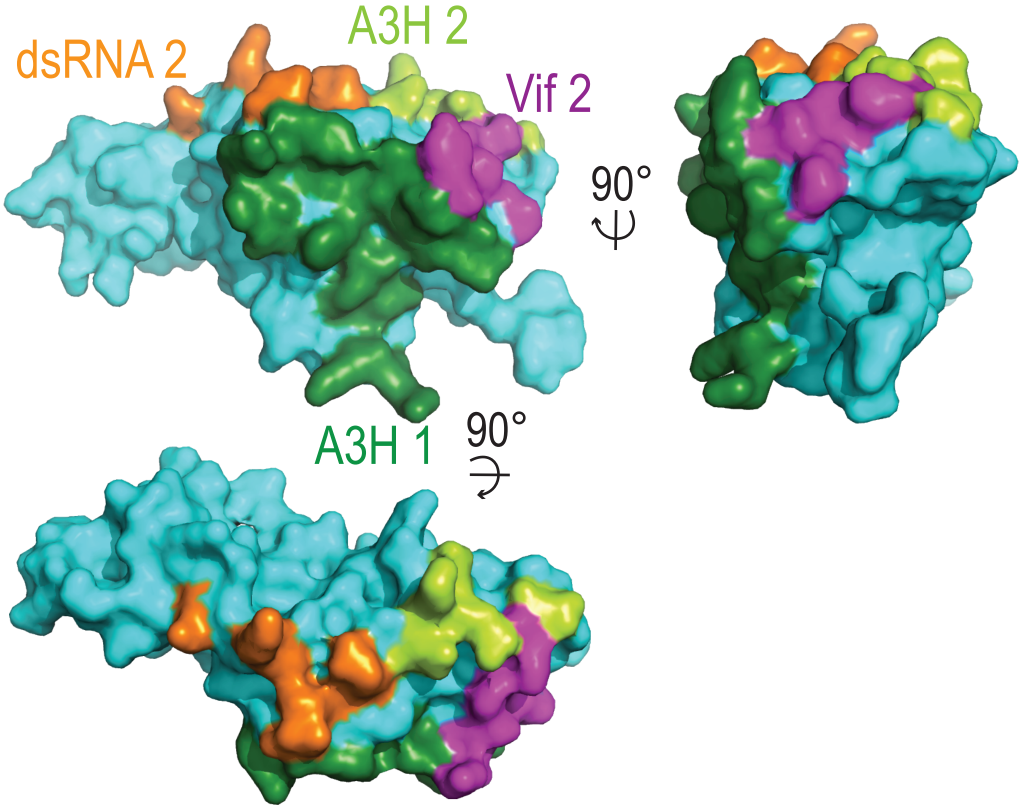
dsRNA 2
A3H 2
Vif 2
90°
A3H 1
90°

Supplementary Figure 11: ***Binding surfaces of Vif*.**
Vif surface residues involved in interactions with cpzA3H from the same protomer (dark green), cpzA3H from the opposing protomer (light green), dsRNA (orange), and the second Vif molecule (purple) are indicated. Interacting surface residues were identified using the *InterfaceResidues* script available on the PyMOLWiki (https://www.pymolwiki.org/index.php/InterfaceResidues).

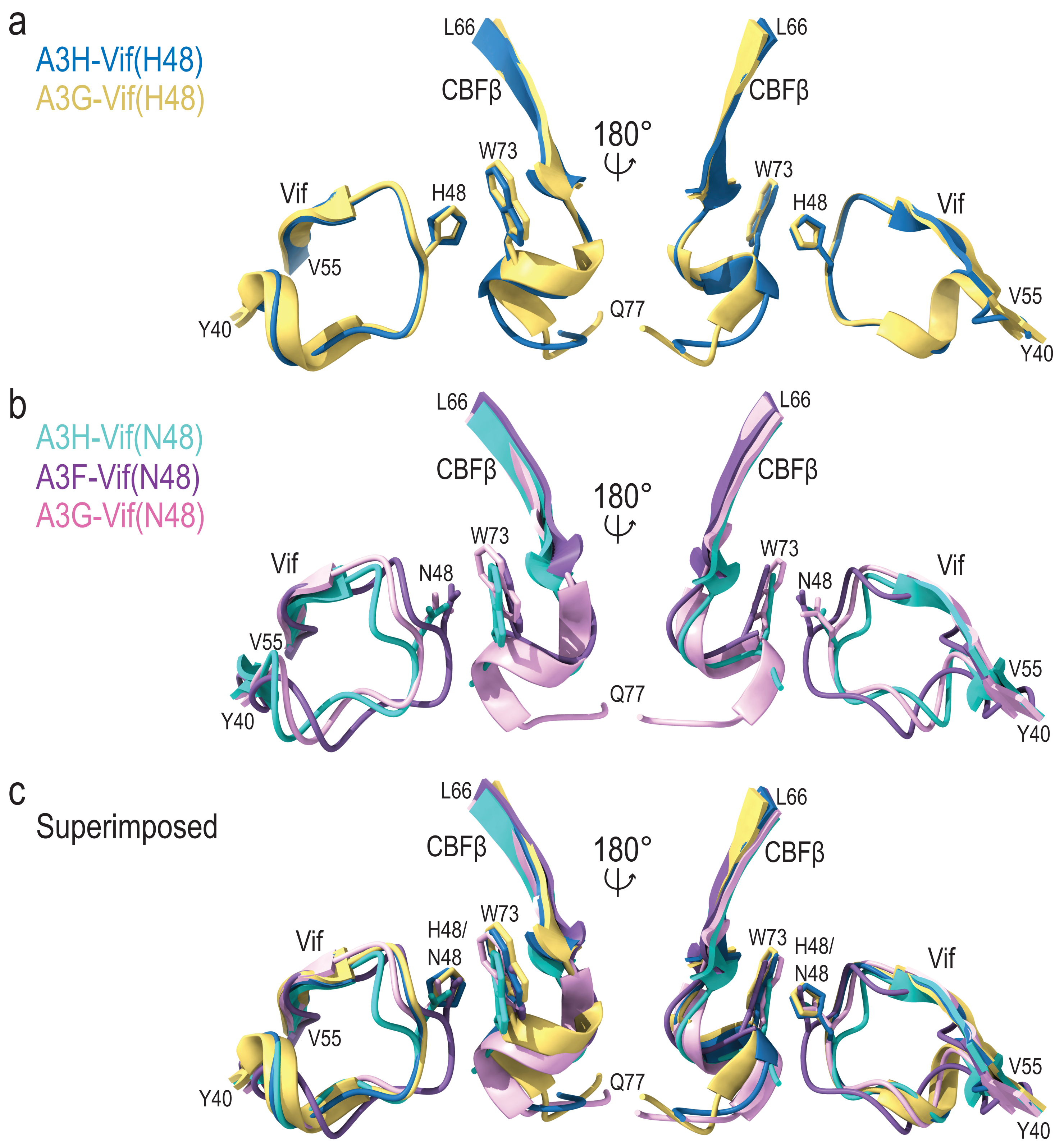
a
A3H-Vif(H48)
A3G-Vif(H48)
L66
CBFβ
180°
W73
H48
Vif
V55
Y40
Q77
b
A3H-Vif(N48)
A3F-Vif(N48)
A3G-Vif(N48)
L66
CBFβ
180°
W73
N48
Vif
V55
Y40
Q77
c
Superimposed
L66
CBFβ
180°
W73
H48/
N48
Vif
V55
Y40
Q77

Supplementary Figure 12 - ***Structural Comparison of Vif H48/N48 Region in Complex with A3F, A3G, and A3H***

Panel **a**: Superimposition of Vif residues 40-55 and CBFβ residues 66-77 from human A3H-VCBC complex (Blue, PDB ID#8FVI)[2], and A3G-VCBC complex (yellow, PDB ID#8CX2)[4]. Both structures feature a histidine at position 48 of Vif (Vif-H48), with W73 of CBFβ being the closest residue to H48. Panel **b**: Superimposition of Vif residues 40-55 and CBFβ residues 66-77 from cpzA3H-VCBC complex (cyan, this study), A3F-VC complex (purple, PDB ID#6NIL)[5], and A3G-VCBC complex (pink, PDB ID#8E40)[6]. All structures feature a Asparagine at position 48 of Vif (Vif-N48), with W73 of CBFβ being the closest residue to H48. Panel **c**: Superimposition of all five structures shown in panels **a** and **b**.

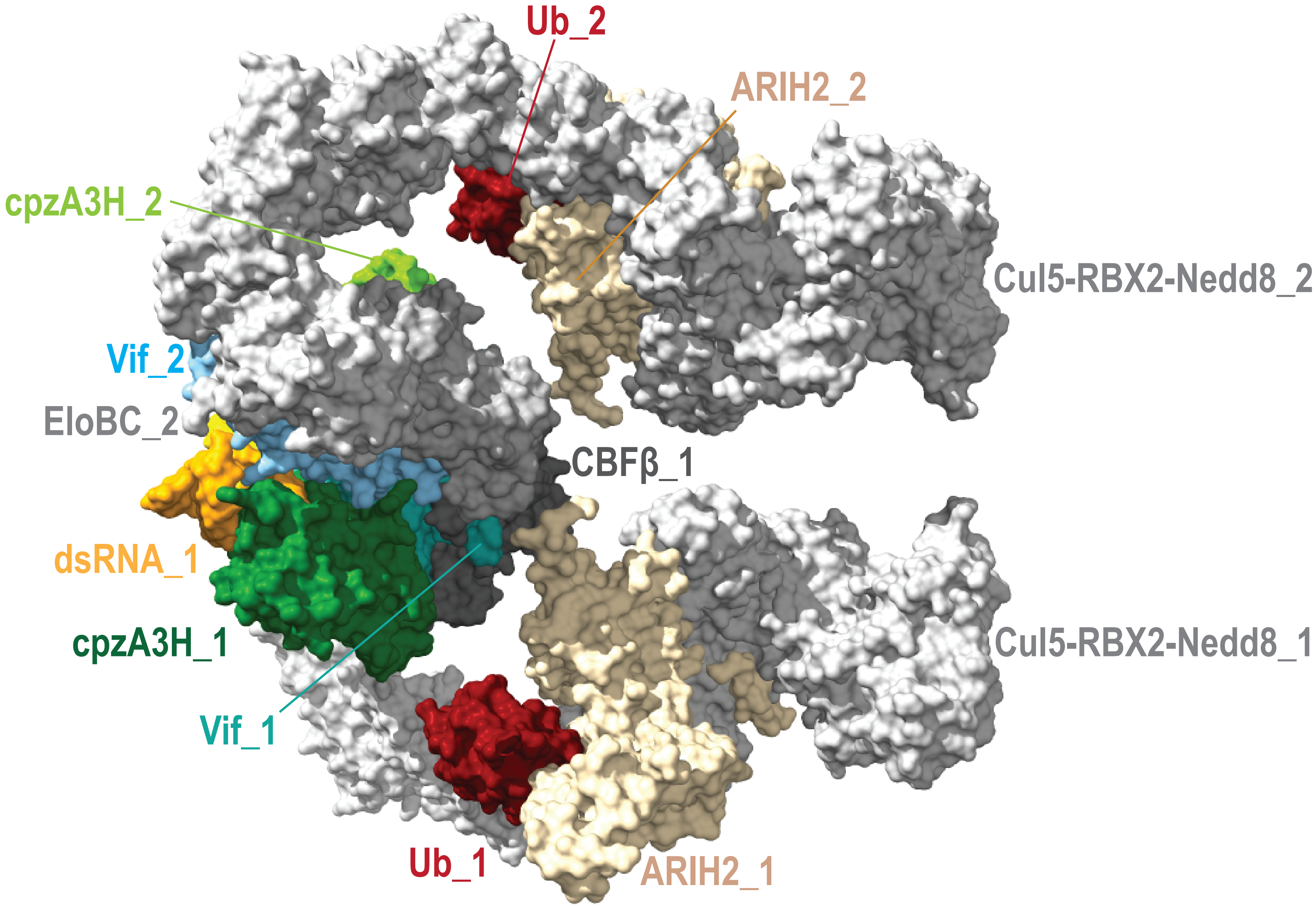
Ub_2
ARIH2_2
cpzA3H_2
Cul5-RBX2-Nedd8_2
Vif_2
EloBC_2
CBFβ_1
dsRNA_1
cpzA3H_1
Cul5-RBX2-Nedd8_1
Vif_1
Ub_1
ARIH2_1

Supplementary Figure 13: ***Structural Model of the dimeric cpzA3H–VCBC complex with two CUL5 E3 Ligase assemblies.***

This model is based on the dimeric cpzA3H–VCBC structure presented in this study, combined with two extended CUL5 E3 ligase complexes. Structural components of the CUL5 E3 ligase complex were incorporated from PDB IDs 4N9F[14], 7B5M[15], and 7ONI[16]. Proteins are color-coded as follows: cpzA3H in green, Vif in cyan, CBFβ in dark gray, EloBC in light gray, components of the CUL5 E3 ligase complex in gray, ARIH2 in beige, and ubiquitin in red. The model depicts two CUL5 E3 ligase complexes, each bound to Vif molecule from each protomer of the cpzA3H–VCBC dimer. Each charged ubiquitin is positioned to engage Vif-proximal cpzA3H molecule, suggesting a plausible arrangement for substrate ubiquitination.

Supplementary References

1. He, J., Li, T. & Huang, S.Y. Improvement of cryo-EM maps by simultaneous local and non-local deep learning. *Nat Commun* **14**, 3217 (2023).
2. Ito, F., Alvarez-Cabrera, A.L., Kim, K., Zhou, Z.H. & Chen, X.S. Structural basis of HIV-1 Vif-mediated E3 ligase targeting of host APOBEC3H. *Nat Commun* **14**, 5241 (2023).
3. Matsuoka, T. et al. Structural basis of chimpanzee APOBEC3H dimerization stabilized by double-stranded RNA. *Nucleic Acids Res* **46**, 10368-10379 (2018).
4. Li, Y.L. et al. The structural basis for HIV-1 Vif antagonism of human APOBEC3G. *Nature* (2023).
5. Hu, Y. et al. Structural basis of antagonism of human APOBEC3F by HIV-1 Vif. *Nat Struct Mol Biol* **26**, 1176-1183 (2019).
6. Ito, F. et al. Structural basis for HIV-1 antagonism of host APOBEC3G via Cullin E3 ligase. *Sci Adv* **9**, eade3168 (2023).
7. Guo, Y. et al. Structural basis for hijacking CBF-beta and CUL5 E3 ligase complex by HIV-1 Vif. *Nature* **505**, 229-33 (2014).
8. Horn-Ghetko, D. et al. Ubiquitin ligation to F-box protein targets by SCF-RBR E3-E3 super-assembly. *Nature* **590**, 671-676 (2021).
9. Kostrhon, S. et al. CUL5-ARIH2 E3-E3 ubiquitin ligase structure reveals cullin-specific NEDD8 activation. *Nat Chem Biol* **17**, 1075-1083 (2021).
