# Supplementary material for: HIV-1 vif mediates ubiquitination of the proximal protomer in the APOBEC3H dimer to induce degradation
Source: Nat Commun. 2025 Jul 1;16:5879. doi: 10.1038/s41467-025-60984-y (PMC12217271; doi:10.1038/s41467-025-60984-y)
Supplement: Supplementary file 2 — Description Of Additional Supplementary File [file 41467_2025_60984_MOESM2_ESM.pdf]

Description of additional supplementary files

Supplementary Data 1

Title: Proteome Discoverer Report

Description: Mass spectrometry results for the identification of ubiquitinated lysines in chimpanzee APOBEC3H (cpzA3H) are presented. This data file contains all identified proteins, peptide sequences, peptide spectrum match counts (PSM#), and MS1 peak areas.
